# Solitary Extramedullary Plasmacytoma of the Thyroid Gland

**DOI:** 10.1155/2012/282784

**Published:** 2012-10-09

**Authors:** M. Ridal, N. Ouattassi, T. Harmouch, A. Amarti, M. N. Alami

**Affiliations:** ^1^ENT Head and Neck Surgery Department, Hassan II University Hospital, Fez 30000, Morocco; ^2^Laboratory of Pathology, Hassan II University Hospital, Fez 30000, Morocco

## Abstract

Solitary extramedullary plasmacytoma of the thyroid gland is an uncommon condition. Up to date, its clinical pathological features are not fully understood. We present a case of an extramedullary nonmucosal plasmacytoma of the thyroid gland which is the first case with regional metastatic lymph nodes. This condition requires a scrupulous survey to rule out a metastatic multiple myeloma. Although localized forms management is still controversial, authors require combined approach for regional metastatic forms. The prognosis is favorable compared to solitary bone plasmacytomas or multiple myeloma.

## 1. Introduction 

Primary plasmacytoma of the thyroid gland is a rare disease. In fact, to our knowledge, only 75 cases of solitary plasmacytoma of the thyroid have been reported in the literature. Some of these cases are poorly documented. Up to date, its clinical pathological features are not fully understood. We report a case of metastatic solitary plasmacytoma of the thyroid gland and discuss the clinical features and management modalities. 

## 2. Case Presentation

**Figure 1:**
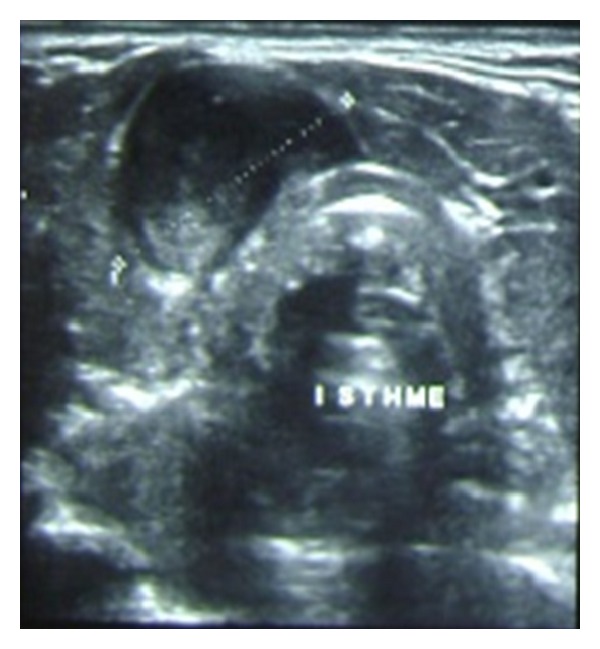
Neck ultrasonography that shows a heterogenous isthmic nodule.

A 52-year-old female patient with no significant medical history was presented to the outpatient clinic with 6 months history of a progressively enlarging painless goiter with no toxic or pressure symptoms. Clinical examination revealed a firm nodular thyroid with a 2-centimeter lymph node of the IVth left cervical area. Neck ultrasonography (Figure 1) confirmed the enlargement of the thyroid gland with the presence of an 18 mm hypervascular isthmic lump and a 20 mm left cervical lymph node. Fine needle aspiration cytology (FNAC) examination of a thyroid node specimen was consistent with a lymphoplasmacytic lymphoma or a plasmacytoma. 

We performed a survey for a multiple myeloma that consisted of an endoscopic examination of the upper aerodigestive tract which had not shown any mucosal lesions. Thoracoabdominal CT scan was normal, with no pulmonary lesions, no mediastinal lymph nodes, and no hepatosplenomegaly. X-ray skeletal survey was also normal.

Laboratory tests were normal, including thyroid function (TSH), serum protein level with no monoclonal gamma globulin peak. Also there was no biological evidence of inflammation, and no Bence-Jones protein was detected. The bone marrow biopsy showed no tumoral proliferation. Antiperoxidase and antithyroglobulin antibodies were negative.

**Figure 2:**
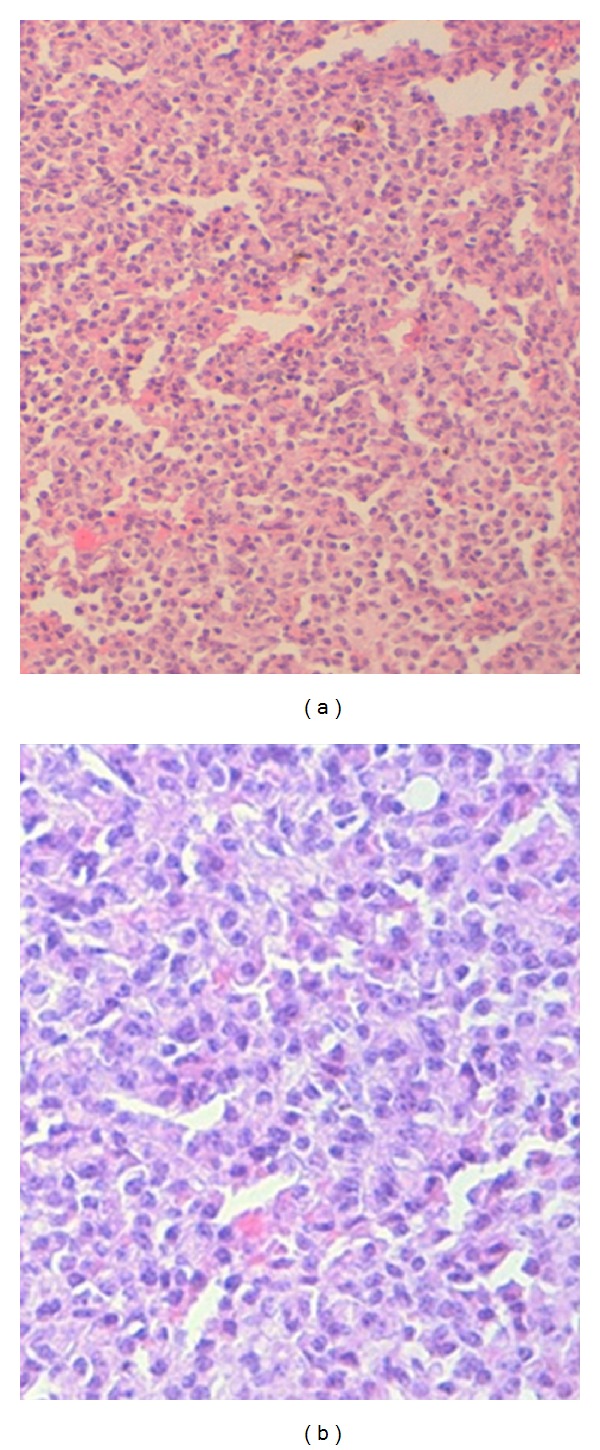
(a) and (b) thyroid parenchyma is infiltrated by a diffuse sheet of neoplastic cells that have an abundant cytoplasm and an eccentric and irregular nucleus.

**Figure 3:**
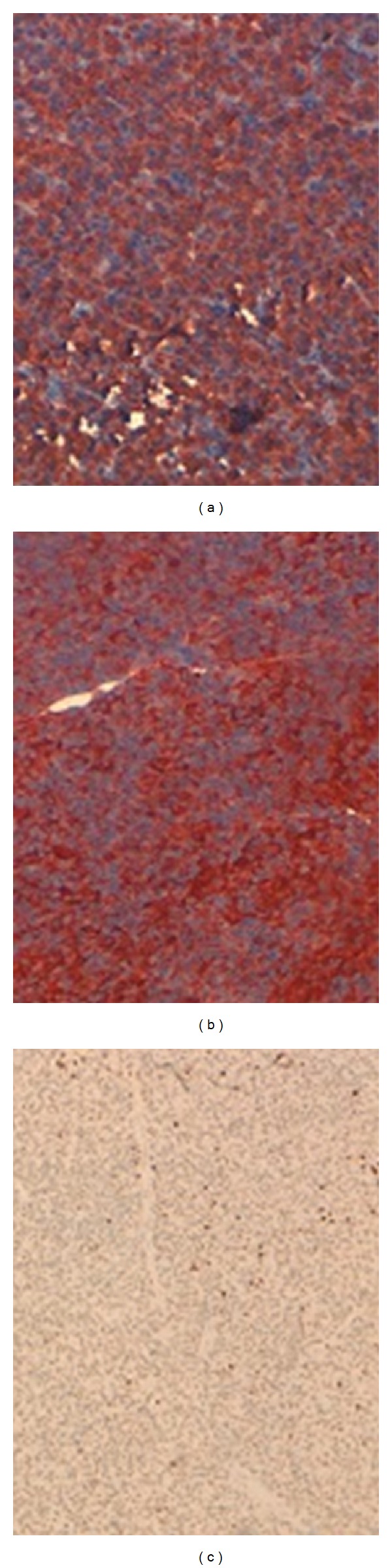
Intense and widespread staining of CD79A (a) and CD 138 (b), Ki 67 very low<10% (c).

The patient underwent a left lobo-isthmectomy with excision of the lymph node. The frozen section examination of the thyroid and the lymph node specimens returned for a lymphomatous process. However, the final pathological examination showed infiltration of thyroid tissue by well-differentiated plasma cells with some immature cells with cytonuclear atypia and high mitotic index (Figures 2 and 3).

The patient underwent a right thyroid lobectomy with mediastino-recurrentiel and cervical functional lymph node bilateral dissection. The postoperative course was uneventful. An additional radiotherapy was performed. The patient remains disease-free at 5 months of followup.

## 3. Discussion

Plasmacytoma is a distinct pool of neoplastic monoclonal plasma cells that can be located in soft tissues or in bone. It belongs to a group of disorders called plasma cell dyscrasias (or monoclonal gammopathies) which includes six major variants that are multiple myeloma, localized plasmacytoma, lymphoplasmacytic lymphoma, heavy-chain disease, primary or immunocyte-associated amyloidosis, and monoclonal gammopathy of undetermined significance. Localized plasmacytomas can occur either in bone (SBP) which can evolve to multiple myeloma or in extramedullary tissues (EMP) which are less than 5% of all plasmacytomas [1]. The most common location of EMP is the upper respiratory tract, oral cavity and salivary glands [1, 2]. The thyroid gland is rarely affected. However, it is not uncommon for multiple myeloma to involve the thyroid gland [1]. 

Three fourths of EMP cases involve males of the 4th to 7th decade [2, 3]. Also, EMP of the thyroid usually presents with painless, firm, mobile, multinodular or diffuse thyroid mass with no associated cervical lymphadenopathy [1]. Rapidly growing thyroid mass that brought the patient to seek medical advice is reported in some series [4]. Out of 195 publications on PubMed regarding the solitary thyroid plasmacytoma, we found no cases of thyroid plasmacytoma with cervical lymph node metastases. We believe this is the first case of metastatic solitary plasmacytoma of the thyroid gland.

Solitary EMP of the thyroid gland is known to occur on a ground of lymphocytic thyroiditis [5]. This has not been true in our case, because the antithyroperoxidase antibodies were negative and there were no histological features of underlying thyroiditis. The diagnosis is made by histology with the unchallenged contribution of the immunohistochemistry. However, the close histogenetic and functional relationship of reticular cells, lymphocytes, and plasma cells reflects the difficulty in classifying lymphoid tumors and distinguishing the various lymphomas of some myelomas. Indeed, the differential diagnosis is not only with lymphoma but also with the medullary and undifferentiated carcinomas [6]. FNAC with research of monoclonal cells expressing kappa light chains allowed the diagnosis in rare cases [7]. One of the most difficult issues in the diagnosis of solitary plasmacytoma of the thyroid is to rule out a multiple myeloma. Thus, normal bone marrow biopsy, the absence of skeletal lytic lesions, and the absence of a monoclonal immunoglobulins peak confirm the diagnosis of solitary thyroid plasmacytoma. Thus, the diagnostic criteria for PEM were established as follows [8]:

- histological evidence of a monoclonal plasma cell infiltration;
- <5% of plasma cells at bone marrow biopsy;
- no skeletal lytic lesions;
- no hypercalcemia or kidney failure;
- low levels of M protein if it exists.

For the tumoral staging, several classifications have been established [9, 10]. However, therapeutic and prognostic value of these classifications requires further evaluation.

The treatment remains controversial. Some authors advocated radiotherapy alone, others advocated surgery alone, while few advocated combined approach [1, 8]. However, for cases of EMP with lymph node involvement or which surgical resection was incomplete or impossible, the combined approach is recommended [11]. Chemotherapy has no role in the treatment of solitary plasmacytomas; it may be interesting in some cases of diffuse disease as part of intensive pretreatment bone marrow transplant.

The prognosis as all localized plasmacytomas remains favorable. Indeed, all sites included, the 10-year overall survival rate is 70%. The rate of progression to multiple myeloma is lower than in SBP, ranging from 11% to 30% at 10 years.Patients with extramedullary plasmacytoma who progressed to multiple myeloma had a 5-year survival rate of 100%, compared with 33% for solitary boneplasmacytoma [11, 12]. 

## 4. Conclusion 

EMP of the thyroid gland is rare. Diagnosis must rule out a multiple myeloma since thyroid involvement in such case is much frequent. The treatment of localized forms is still controversial. However, authors are unanimous about the combined approach in regional metastatic forms. The prognosis remains favorable compared to solitary bone plasmacytomas.
